# Friend vs. Foe: Cognitive and Affective Empathy in Women With Different Hormonal States

**DOI:** 10.3389/fnins.2021.608768

**Published:** 2021-03-08

**Authors:** Ann-Christin Sophie Kimmig, Dirk Wildgruber, Sina-Maria Ute Wendel, Inger Sundström-Poromaa, Birgit Derntl

**Affiliations:** ^1^Department of Psychiatry and Psychotherapy, University of Tübingen, Tübingen, Germany; ^2^International Max Planck Research School for Cognitive and Systems Neuroscience, University of Tübingen, Tübingen, Germany; ^3^Department of Women’s and Children’s Health, University of Uppsala, Uppsala, Sweden; ^4^LEAD Graduate School and Research Network, University of Tübingen, Tübingen, Germany; ^5^Tübingen Neuro Campus, University of Tübingen, Tübingen, Germany

**Keywords:** empathy, cognitive empathy, affective empathy, emotional closeness, hormonal status, oral contraceptives

## Abstract

Empathy is crucial for social functioning as well as social coherence. It can be influenced by modulatory factors such as familiarity and liking (i.e., emotional closeness). Furthermore, there are first hints that hormonal status may modulate affective but not cognitive empathy in women. The aim of this study was to investigate potential separate as well as combined modulatory effects of emotional closeness and hormonal status on female cognitive and affective empathy. Three hormonal status groups of women (*n* = 62) were studied: (1) naturally-cycling (NC) women in the early follicular phase (fNC), (2) NC women during periovulatory phase (oNC), and (3) oral contraceptive (OC) users. All women underwent a newly developed empathy task (i.e., Tübinger Empathy Test, TET) presenting textual descriptions of positive and negative emotional scenes relating to three different perspectives (i.e., self vs. friend vs. enemy/disliked person). Regardless of hormonal status, empathic responses were higher for the friend compared to the enemy perspective for both empathy components. However, cognitive empathy was less affected by varying emotional closeness toward the target person than affective empathy. Hormonal status modulated only affective empathy. OC users showed significantly less affective empathy toward the enemy compared to the fNC women. Overall, affective empathy seems more sensitive to modulatory effects of emotional closeness and hormonal status than cognitive empathy. Possible implications of this current investigation for future research on empathy and OC use, contraceptive education as well as for other clinical applications are discussed.

## Introduction

Correctly inferring emotional states and intentions through the observation of others’ behavior is a prerequisite for successful social interaction and strengthens social coherence (de Vignemont and Singer, 2006). Typically, three core components defining empathy are derived (Decety and Jackson, 2004) namely (1) the ability to recognize emotions via non-verbal cues (i.e., facial expressions, gesture, body posture, gait, speech prosody, etc.), (2) an affective component enabling the experience or sharing of similar emotions with others and (3) a cognitive component describing the ability to infer the emotional states of others, even in the absence of non-verbal cues and pronounced affective responses. Even though these different components work independently to some extent, ultimately, they cannot be completely disentangled and rely partly on each other (Zaki and Ochsner, 2012). It is assumed that in generating the cognitive as well as affective component of empathy, self-related simulation processes such as self-projection are involved (Gordon, 1986; Heal, 1996; Decety and Grèzes, 2006; Waytz and Mitchell, 2011; Zaki and Ochsner, 2012). Self-projection is defined as predicting the mental state of a target person by imagining oneself in the respective situation and generalizing one’s own thoughts and feelings to the other person.

There is first evidence that empathy-related processes such as self-projection among others are influenced by the familiarity and liking toward the target person (Engert et al., 2014; Bucchioni et al., 2015; Meyer et al., 2015). In Engert et al.’s (2014) study participants watched either a loved person or a stranger through a one-way mirror or a video transmission undergoing the Trier Social Stress Task (TSST), in which the target person has to perform a mock job interview and demanding mental arithmetic in front of an evaluation committee. During the TSST, cortisol levels of participants as well as the target persons were measured. They found that participants exhibited significantly increased empathic stress (i.e., cortisol increase) when watching their loved person compared to a stranger. While this study focused on the affective empathy component, Bucchioni et al. (2015) used a perspective taking task, thus tapping into the cognitive empathy component. During this perspective taking task, participants were shown images of painful situations and were asked to either imagine this happening to (a) themselves, (b) the most familiar loved person, (c) the most familiar hated person and (d) a stranger and rate the pain experienced by that target person. Pain ratings for the familiar loved person were significantly higher than for all other perspectives. Moreover, the response times were significantly shorter for the self and the familiar loved person (Bucchioni et al., 2015). Overall, these studies suggest not only an increased but also a facilitated empathic response as a function of familiarity and liking. Therefore, familiarity and liking toward a target person seem to play a role in cognitive as well as affective empathic responsiveness and should be accounted for when evaluating empathic abilities. Since it is difficult to disentangle the effects of familiarity and liking, as one seldomly stays constant when the other changes, we refer to the combination of both factors as emotional closeness from now on. No study has yet compared the extent of emotional closeness effects between cognitive and affective empathy. Therefore, it is not clear whether both empathy components are affected equally by changes in emotional closeness toward the target person. Furthermore, there is not much known whether empathic responses toward target persons with varying emotional closeness are modulated differently by other factors (e.g., sex, hormonal status, cognitive functioning, or psychopathology), thus including multiple target persons with different levels of emotional closeness in a study design is likely to give a more comprehensive picture of a person’s general empathic abilities.

With regards to hormonal status, evidence is accumulating that female sex hormones like estradiol and progesterone (i.e., endogenous as well as synthetic) affect a variety of socio-emotional processes such as mood, fear processing, and sexual desire as well as arousal (Montoya and Bos, 2017; Lundin et al., 2018; Lewis et al., 2019). It has been suggested that menstrual cycle dependent as well as OC-induced changes in mental states and behavior may be explained by a differential binding of endogenous (i.e., due to changing concentrations) as well as synthetic sex steroids (i.e., due to slightly different binding properties) to receptor sites of brain regions involved in socio-emotional processing (e.g., limbic areas and frontal cortex; Toffoletto et al., 2014; Barth et al., 2015; Louw-du Toit et al., 2017). This differential binding and the resulting changes in transcription cascades can lead to hormone-induced changes of neural activity as well as brain morphology (Toffoletto et al., 2014; Barth et al., 2015; Rehbein et al., 2021). Due to these modulatory effects of hormonal status on brain regions relevant for socio-emotional processing, it would not be surprising if next to influences on mood, fear processing and sexual desire (Montoya and Bos, 2017; Lundin et al., 2018; Lewis et al., 2019), hormonal status also affected empathy.

Most commonly the association between the sex hormone testosterone and empathy has been investigated so far. In females, testosterone has been negatively associated with empathic processes such as perspective taking (Nitschke and Bartz, 2020) and complex emotion recognition (van Honk et al., 2011; Bos et al., 2016). Furthermore, more utilitarian moral judgments of women after being administered synthetic testosterone indicate a blunting effect on empathy (Montoya et al., 2013; Chen et al., 2016). Regarding female sex hormones, a number of studies have investigated the effect of estradiol and progesterone on emotion recognition, however evidence on modulatory effects of hormonal states on the cognitive and affective components of empathy is scarce (for review see: Montoya and Bos, 2017). Previous studies suggest an hormone-related modulatory effect on emotion recognition (Pearson and Lewis, 2005; van Wingen et al., 2007; Derntl et al., 2008; Guapo et al., 2009; Derntl et al., 2013; Hamstra et al., 2014; Kamboj et al., 2015; Pahnke et al., 2019) and affective responsiveness, but not perspective-taking (Derntl et al., 2013; Radke and Derntl, 2016). In these studies (Hamstra et al., 2014; Pahnke et al., 2019), OC use was linked to a reduced emotion recognition performance compared to naturally cycling (NC) women. Furthermore, progesterone (van Wingen et al., 2007; Derntl et al., 2008, 2013) and estradiol levels (Pearson and Lewis, 2005; Guapo et al., 2009; Kamboj et al., 2015) have been negatively correlated to emotion recognition of negative emotions. Affective responsiveness performance was modulated by menstrual cycle phase (follicular vs. midluteal; Derntl et al., 2013) as well as OC-phase (active vs. pill-break; Radke and Derntl, 2016). Progesterone was shown to be positively correlated to affective responsiveness in NC women, whereas no such association was found for estradiol levels (Derntl et al., 2013). Thus, hormonal status seems to have a modulatory effect on at least some empathy-related processes. However, most studies so far have been limited to tapping only into one empathy component, and no study has included target persons with different levels of emotional closeness. Given the tremendous number of OC users worldwide and the crucial role of empathy in social interaction (de Vignemont and Singer, 2006) it is highly relevant to investigate potential effects of OC-intake on empathy more closely not only for millions of OC users, but also for their social contacts and ultimately for society.

Therefore, the main aim of this study was to systematically investigate potential modulatory effects of hormonal status on cognitive and affective empathic responses toward target persons with varying emotional closeness in healthy women. For this purpose, we designed a new empathy task (i.e., the Tübinger Empathy Test, TET) using verbal descriptions of emotional situations to tap into cognitive as well as affective empathy toward two target persons (i.e., perspectives) with varying emotional closeness (i.e., friend and enemy). Next to the inclusion of target persons with differing levels of emotional closeness, the TET was developed to tackle some shortcomings of already existing empathy paradigms and thus to capture a more comprehensive and accurate picture of empathy. For instance, the TET contains an equal number of positive and negative emotion conditions (i.e., three per valence) to represent a broad spectrum of different emotions while avoiding a bias toward negative emotions, which is present in most existing empathy research (Motomura et al., 2015; Kogler et al., 2020). Furthermore, verbal descriptions of emotional situations were chosen to avoid confounding effects of sensory aspects of emotion recognition (which has been shown to be affected by hormonal status) as well as emotional matching. These confounding effects are hard to deal with in picture/video-based tasks or in real-life paradigms, which have been recently suggested by Shamay-Tsoory and Mendelsohn (2019). To test potential modulatory effects of different hormonal states, we included naturally cycling women during the early follicular phase (fNC, characterized by low estradiol and low progesterone levels) or the periovulatory phase (oNC, characterized by high estradiol and low progesterone levels) and women with long-term OC-use [OC, characterized by low levels of endogenous estradiol and progesterone, but high levels of progestogens (Lovett et al., 2017)]. The early follicular phase was chosen to control for the added effects of synthetic sex hormones (i.e., especially progestogens) in OCs as in both groups endogenous sex hormones are low. The inclusion of the periovulatory phase, on the other hand, allowed to test for potential estradiol driven effects. Since this task was employed for the first time, we were also interested whether independent of hormonal status we can replicate the emotional closeness effects found in previous studies and expanding these finding by studying both, cognitive and affective empathy, and contrasting the emotional closeness effects between these two empathy components directly.

Regarding hormone-independent modulatory effects of emotional closeness, we expected.

1. Significantly higher empathic responsiveness as well as a facilitated response (i.e., faster response times) for the friend vs. the enemy perspective, irrespective of empathy component (Engert et al., 2014; Bucchioni et al., 2015) and
2. Cognitive empathy being less sensitive to changes in emotional closeness than affective empathy, due to its more rational nature through the use of reasoning (Einolf, 2012; Zaki and Ochsner, 2012).

Regarding modulatory effects of hormones, preliminary findings suggest that hormonal status influences affective responsiveness, but not perspective taking (Derntl et al., 2013; Radke and Derntl, 2016). Therefore, we hypothesized that the

3. Hormonal status affects the affective rather than the cognitive empathy component.

Up to now, there is no study which has investigated modulatory effects of hormonal status on empathic responsiveness toward target persons with varying levels of emotional closeness (i.e., familiarity and liking). Therefore, the investigation of a possible interplay of emotional closeness and hormonal status on empathic responses was explorative.

## Materials and Methods

### Sample Description

In total, 67 healthy female students of the University of Tübingen were recruited via university round mail. Three participants were excluded from analysis as an LH surge could not be detected in the defined time frame. Another two participants were excluded due to progestogen-only contraception and recent switch of OC brand. The remaining participants (*n* = 62) were divided into three hormonal status groups: (1) women with long-term (> one year) OC-use (OC group; *n* = 22, *m*_age_ = 22.1 ± 2.0), (2) NC women during the early follicular phase (fNC group; *n* = 20, *m*_age_ = 22.3 ± 2.8), and (3) NC women during their periovulatory phase (oNC group; *n* = 20, *m*_age_ = 23.6 ± 3.7). The sample size (*n* = 62) was based on previous, conceptually-related studies (Derntl et al., 2013; Radke and Derntl, 2016; Dan et al., 2019; Gurvich et al., 2020).

General inclusion criteria for this study entailed: 18–35 years of age, no history of any neurological or psychiatric disorders and no (other) hormonal treatment within the past 3 months. For the OC group, all combined OCs were monophasic and minimum intake duration was 6 months (mean duration: 3.3 years ± 1.7 years). Participants in the OC group were only measured in their active pill intake phase (from day 2–21). Only NC women with an average cycle length of 21–35 days and no hormonal contraception for at least the past 6 months were included. oNC women were measured in their fertile period spanning from 3 days prior to 3 days after the detection of the LH peak. The test results were validated with the reported starting date of their menstruation after measurement. Women in fNC group were measured between day 2 and 5 of their menstruation. The women in the hormonal status groups were matched for age, verbal intelligence, and executive functioning. An overview of these sociodemographic and neuropsychological characteristics and the serum hormone profiles for the different hormonal status groups is provided in Table 1.

**TABLE 1:** Sample description (mean and standard deviation if not otherwise specified) and hormone profiles per hormonal status group.

Hormonal status group	OC	fNC	oNC	*p*-value
*N*	22	20	20	
Age (years)	22.1 (2.0)	22.3 (2.8)	23.6 (3.7)	0.32
Verbal IQ (raw scores)	31.8 (3.2)	32.5 (2.4)	31.7 (3.6)	0.69
TMTB-A (sec)	15.5 (9.5)	15.1 (13.5)	16.2 (12.5)	0.96
Trait empathy	44.4 (7.0)	46.1 (5.5)	45.1 (6.6)	0.68
Fantasy	14.4 (3.9)	15.6 (3.1)	14.1 (3.2)	0.37
Perspective-taking	14.5 (3.9)	15.9 (3.0)	16.2 (2.2)	0.17
Empathic concern	15.5 (2.4)	14.7 (3.2)	14.8 (2.6)	0.58
Personal distress	10.3 (3.4)	9.5 (2.7)	8.5 (2.3)	0.12
State anxiety	34.9 (9.6)	33.2 (4.8)	33.1 (7.0)	0.99
Positive mood (PANAS)	31.9 (6.0)	31.2 (6.2)	30.3 (7.2)	0.72
Negative mood (PANAS)	13.8 (4.2)	12.9 (4.0)	12.7 (4.6)	0.44

**Hormone profiles**	**Median (IQR)**	**Median (IQR)**	**Median (IQR)**	

Estradiol (pmol/l)	56.5 (44.0)	159.0 (63.0)	417.5 (254.0)	<0.001 oNC> fNC > OC
Progesterone (nmol/l)	1.3 (0.7)	2.1 (1.3)	2.0 (10.7)	0.001 fNC = oNC > OC
Testosterone (nmol/l)	0.8 (0.3)	1.1 (0.4)	1.3 (0.5)	<0.001 fNC = oNC > OC
SHBG (nmol/l)	160.0 (200.0)	59.0 (47.0)	47.0 (33.0)	<0.001 OC > fNC = oNC

### Procedure

**FIGURE 1:**
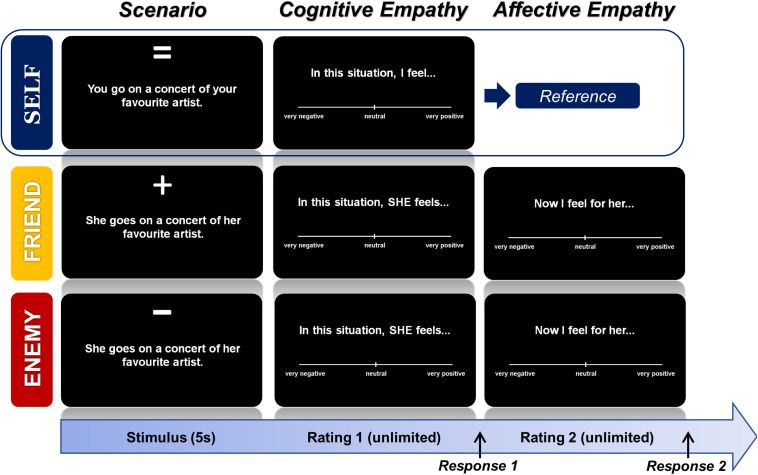
Empathy task design—Tübinger Empathy Test (TET). Each emotional scenario (textual depiction) was presented from three perspectives: the self, friend, and enemy. For each perspective, there were six items per emotion (i.e., happiness, sexual pleasure, gratefulness, anger, disgust, and fear). After a fixed duration of 5 s (s), participants gave a cognitive rating (i.e., how the target person would feel in such a situation) on a visual analog scale ranging from very negative to very positive. The rating time was unlimited. After a response is given a new trial starts in the self-perspective block, whereas in the friend and enemy-perspective block a second, unlimited rating for the affective component (i.e., how does the participant feel, when this happens to the target person) was presented before a new trial started. The self-ratings were used as a predictor for the cognitive and affective ratings of the other two perspectives in order to compute empathic responsiveness in the form of standardized regression coefficients.

After completing the informed consent form, all participants underwent a screening to control for inclusion and exclusion criteria including questionnaires regarding menstrual cycle and OC history, gynecological history (e.g., premenstrual syndrome—PSST; Bentz et al., 2012), pregnancies, endometriosis, polycystic ovary syndrome etc.), verbal intelligence (WST; Schmidt and Metzler, 1992), executive functioning (TMT; Reitan, 1992) and the German version of the Structured Clinical Interview (SCID; Wittchen et al., 1997) to exclude a history of mental illness. The measurement session included (1) a battery of different questionnaires including the assessment of mood using the Positive and Negative Affect Scale (PANAS; Watson et al., 1988), levels of state anxiety (STAI; Laux et al., 1981) and trait empathy (i.e., German version of interpersonal reactivity index (IRI) called SPF; Paulus, 2009), (2) the TET (see Figures 1), and (3) a blood withdrawal for hormonal analyses. Next to the TET, tasks concerning approach and avoidance behaviour (AAT) as well as reward sensitivity were performed. However, results of these will be reported elsewhere. The data collected from the various questionnaires was used to control for potentially confounding effects of mood, state anxiety and trait empathy differences among the hormonal status groups on the behavioral empathy measures. The study was approved by the Ethics committee of the Medical Faculty of the University Tübingen.

#### Tübinger Empathy Test (TET)

All women underwent a newly developed empathy task (i.e., the Tübinger Empathy Test), which presents textual descriptions of real-life emotional scenes (i.e., *positive emotions*: happiness, gratefulness, sexual pleasure, and *negative emotions*: anger, fear and disgust) adapted from a former study (Derntl et al., 2009) relating to three different perspectives (i.e., self vs. friend vs. enemy/disliked person) with varying emotional closeness. All items have been pre-validated. A short description of the pre-validation of items and ratings regarding the valence, arousal, and dominance of the emotional scenes under a self-perspective is presented in Supplementary Material to provide more general information on the stimuli set used. Examples for the real-life, relatable emotional scenes are for instance: “Driving down a hill, your/her brakes stop working.” “You/she miss/es the train, a friend drives you/her to your/her appointment,” “With every gentle touch you/she become/s more aroused.”

The self-perspective was used to operationalize the self-projection aspect (Gordon, 1986; Heal, 1996; Decety and Grèzes, 2006; Waytz and Mitchell, 2011; Zaki and Ochsner, 2012) in the measures of empathy by using it as a predictor for ratings of (1) the emotional valence the other person would experience in the same situation (cognitive component) and (2) their own emotional valence when the other person is experiencing the described situation (affective component). Moreover, this approach also allows to control for other potentially confounding factors such as alexithymia (i.e., the inability to recognize and/or describe one own’s emotional states; Jonason and Krause, 2013). For the friend and enemy perspectives, participants were instructed to think of a specific person fulfilling a certain set of characteristics (e.g., for friend: trustworthy, reliable and fun; for enemy: disliked/arrogant, conflict-laden relationship, wanting to keep distance). Participants’ task was to imagine living through the described scenario and to rate how the respective person would feel (on a dimensional scale from positive to negative) in such a situation and which feeling she experiences herself, while imagining the other person in such a situation (see Figure 1).

The sentences were shown for 5 s followed by a fixation cross (250 ms) and visual analogue scales (VAS) to assess emotional ratings (i.e., one VAS for *self-*condition; two VAS for each *other-*condition, separated by 250 ms lasting fixation crosses), which could be operated using computer mouse movements and button presses. Each trial was followed by an 1 s fixation cross. Six trials per condition were presented for all three perspectives (6 × 6 × 3 = 108 trials). The sentences were presented in six blocks of 18 items, each block consisting of items from the same perspective and the same valence of emotion (i.e., positive or negative block). The sequence of items within the blocks was randomized for each participant. Ratings from -100 (very negative) to + 100 (very positive) and response times were recorded. Even though participants were instructed to give swift responses, they had no time restriction to enter their ratings. The task lasted about 20 min.

#### Hormone Sampling and Analyses

To confirm cycle phase as well as inter-individual differences in sex steroid concentrations, blood levels of estradiol, progesterone, testosterone, and the sex hormone binding globulin (SHBG) were analyzed. Per participant, two 7.5 ml serum monovettes were used for blood draw. After blood collection, the samples were immediately sent to the university clinic’s laboratory (“Zentrallabor Universitätsklinik Tübingen”). There the samples were analyzed using chemiluminescence immunoassays (CLIA; Centaur, Siemens; more detailed information in Supplementary Material). For all serum hormone concentrations, the measurement units were nmol/l except for estradiol, which was measured in pmol/l.

### Data Analysis

Data analysis was carried out with the statistical software IBM SPSS Statistics 25 (IBM, New York). If not otherwise specified, two tailed testing was carried out with an α–level of .05. In addition to test statistics and *p*-values, test-appropriate effect sizes are reported.

#### Demographic Information and Hormone Concentrations

To check for potentially confounding effects of sociodemographic and personality factors on the empathy measures, age, verbal intelligence, executive functioning, and empathy trait measures as well as mood and state anxiety at baseline were analyzed for group differences.

A multivariate ANOVA was run to determine whether there are any significant hormonal status (OC, fNC, oNC) differences in age, verbal intelligence (WST score), executive functioning (TMT-B time minus TMT-A time), trait empathy (IRI score for total empathy and the four subscales), positive affect (PANAS) and testosterone levels. Welch’s ANOVA was reported when the assumption of variance homogeneity was violated. In addition, potential differences in hormonal levels of endogenous estradiol, progesterone, and SHBG as well as state anxiety (STAI) and negative affect (PANAS), which were all not normally distributed, were analyzed using the non-parametric Kruskal-Wallis ANOVA. In case of a significant main effect of hormonal status, multiple comparison corrected Bonferroni *post hoc* analyses were carried out to disentangle this effect.

#### Tübinger Empathy Test (TET)

We expressed cognitive empathy and affective empathy as individual standardized regression coefficients (beta values). Cognitive empathy was computed for every participant using a regression analysis with the ratings of their emotional valence when being in the various situations themselves as the predictor of their ratings of the emotional valence the other person would experience in the same situation (friend or enemy, respectively). Affective empathy was calculated on the basis of a further regression analysis using the emotional valence ratings under the self-condition as the predictors of the ratings of their own emotional valence when the other person is experiencing the described situation (friend or enemy, respectively). For calculating these regression weights, only raw scores, for which response times did not exceed three standard deviations of the individual’s mean of the respective perspective condition (i.e., mean drop-out about 2%), were used. Using standardized regression weights for the conceptualization of empathic responsiveness was chosen to (1) control for any inter-individual differences in experiencing such situations *per se*, (2) lean on the previously described concept of self-projection in empathy and (3) aid comparison of outcomes with other studies by using a standardized measure.

Since the cognitive and affective component have been reported as largely independent (Decety and Jackson, 2004), however, not completely dissociable from each other (Zaki and Ochsner, 2012), we decided to include both components in the same mixed ANOVA analysis. Therefore, the respective individual standardized regression coefficients for each participant as well as the response times were subjected to two separate mixed-effects ANOVA including the within-subject factors empathy component (cognitive, affective) and perspective (friend, enemy) and the between-subjects factor hormonal status (OC, fNC, oNC). Due to equal sample sizes and the consequently relative large robustness of ANOVA to non-normally distributed data (Blanca et al., 2017), it was decided to carry on with this analysis even if the dependent variables were not completely normally distributed. *Post hoc* testing was controlled for multiple testing using Bonferroni correction. When data for *post hoc* testing was non-parametric, Welch’s ANOVA and Games-Howell testing or Wilcoxon tests were used instead.

Correlational analyses of self-reported with task-related empathy measures are reported in Supplementary Material.

## Results

### Demographics and Hormone Concentrations

In order to test whether empathic responsiveness is stable across different hormonal states, the hormonal status groups (i.e., OC: *n* = 22, fNC: *n* = 20 and oNC: *n* = 20) were matched for possible confounders including demographic variables such as age, verbal intelligence, and executive functioning to exclude potentially confounding factors (see Table 1). The hormonal status groups also did not differ for trait empathy as well as baseline mood (PANAS) and state anxiety prior to measurement. Table 1 also shows that hormone concentrations varied as expected across the hormonal phases in which the women were examined.

### Empathic Responsiveness: Role of Empathy Component, Perspective, and Hormonal Status

Results of the mixed-effects ANOVA (empathy component × perspective × hormonal status) revealed significant main effects of empathy component [*F*(1, 59) = 240.39, *p* < 0.001, *_p_*η^2^ = 0.80, 95% CI (0.40, 0.52)], and perspective [*F*(1, 59) = 226.70, *p* < 0.001, *_p_*η^2^ = 0.79, 95% CI (0.36, 0.47)]. Empathic responses were significantly higher for the cognitive compared to the affective component (*post hoc t*-tests, all *p* < 0.001), and friend-related empathic responses were significantly higher than enemy-related responses (*post hoc t*-tests, all *p* < 0.001). Furthermore, the interaction of empathy component^∗^perspective was significant [*F*(1, 59) = 181.76, *p* < 0.001, *_p_*η^2^ = 0.76]. A *post hoc t*-test revealed that the difference of affective empathy for friend vs. enemy was significantly larger than in cognitive empathy, which was paralleled by a significantly larger gap between cognitive and affective empathy in the enemy than the friend perspective [*p* < 0.001, *r* = 0.87, 95% CI (-0.88, -0.64)].

**FIGURE 2:**
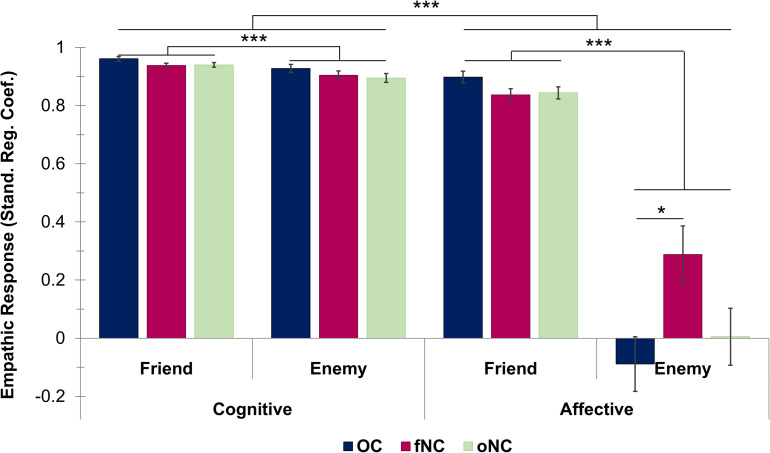
Bar chart depicting the empathic responsiveness (measured by standardized regression coefficients) for cognitive empathy (left half) and affective empathy (right half) for each hormonal status group [i.e., OC—oral contraceptive users (blue), fNC—naturally cycling women in early follicular phase (magenta) and oNC—naturally cycling women in periovulatory phase (light green)] divided up into the friend and enemy perspectives. Error bars with 1 SE. *p < 0.05, ***p < 0.001.

We found no general effect of hormonal status on empathic responsiveness [*F*(2, 59) = 2.26, *p* = 0.11]. However, all interaction terms of hormonal status and the within-factors [empathy component^∗^hormonal status: *F*(2, 59) = 3.27, *p* = 0.05, *_p_*η^2^ = 0.10; perspective^∗^hormonal status: *F*(2, 59) = 5.52, *p* = 0.006, *_p_*η^2^ = 0.16; empathy component^∗^perspective^∗^hormonal status: *F*(2, 59) = 5.29, *p* = 0.008, *_p_*η^2^ = 0.15] were significant. Disentangling of the three-way interaction revealed hormonal status effects only in the difference of perspectives (i.e., friend—enemy) for affective empathy [*F*(2, 59) = 5.46, *p* = 0.04, η^2^ = 0.16], with the fNC group having a smaller difference than the OC group [*p* = 0.006, 95% CI (-0.76, -0.11)]. This difference is driven by a significantly larger affective empathy toward the enemy in the fNC than the OC group [*p* = 0.02, 95% CI (0.04, 0.71); hormonal status effect: *F*(2, 59) = 4.11, *p* = 0.04, η^2^ = 0.12, see Figure 2]. Hormonal status groups did not differ for friend-related affective empathy [*F*(2, 35.84) = 3.30, *p* = 0.10]. All remaining *post hoc* analyses remained non-significant (all |*F*| ≤ 4.34, all *p* ≥ 0.07). The oNC group did not differ significantly from any other hormonal status group (all *p* ≥ 0.10).

### Response Times: Role of Empathy Component, Perspective, and Hormonal Status

**FIGURE 3:**
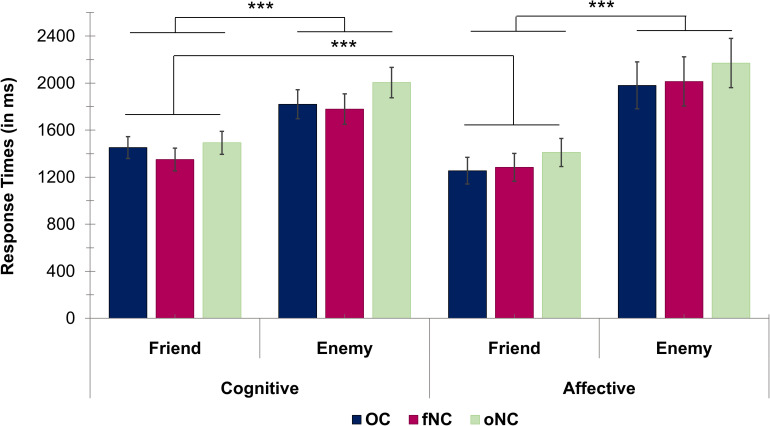
Bar chart depicting the response times (in ms) for cognitive ratings (left half) and affective ratings (right half) for each hormonal status group [i.e., OC—oral contraceptive users (blue), fNC—naturally cycling women in early follicular phase (magenta) and oNC—naturally cycling women in periovulatory phase (light green)] divided up into the friend and enemy perspectives. Error bars with 1 SE. ***p < 0.001.

Results of the mixed-effects ANOVA (empathy component × perspective × hormonal status) of the response times revealed no significant main effect of the empathy component [*F*(1, 59) = 0.48, *p* = 0.49]. Perspective, however, was significant with quicker responses for the friend compared to the enemy perspective [*F*(1, 59) = 149.08, *p* < 0.001, *_p_*η^2^ = 0.72, 95% CI (-683.72, -491.19), see Figure 3]. The interaction empathy component^∗^perspective was significant [*F*(1, 59) = 21.93, *p* < 0.001, *_p_*η^2^ = 0.27]. The response time difference for friend vs. enemy was significantly larger for the affective than the cognitive component (*p* < 0.001, *r* = 0.54). *Post hoc* analyses revealed for friend-related ratings significantly faster response times for the affective than cognitive component (*p* = 0.001, *r* = -0.46), whereas no significant difference emerged for the enemy perspective (*p* = 0.23).

We found no general effect of hormonal status on empathic responsiveness [*F*(2, 59) = 0.49, *p* = 0.62] nor an interaction effect (all |*F*| < 0.33, all *p* ≥ 0.72).

## Discussion

The main aim of this study was to thoroughly investigate potential modulatory effects of hormonal status on the different empathic components (i.e., cognitive and affective) using different target persons with varying levels of emotional closeness in an otherwise homogenous female sample. For this purpose, the newly designed TET was used as it integrates several target persons with varying emotional closeness (i.e., perspectives: friend vs. enemy) and uses an equal number of positive and negative emotional scenarios. Therefore, providing comprehensive measures of cognitive and affective empathic responsiveness. Furthermore, since this is the first time the task was used, we were interested whether we can replicate and extend previous findings regarding hormone independent effects of emotional closeness. Regarding the pre-validation of the TET, the majority of stimuli were adapted from the already well-established affective responsiveness task by Derntl et al. (2009). In addition, only items with recognition accuracy levels higher than 80 percent (chance-level at about 9 percent) and unambiguous valence ratings were selected after a small pre-validation study (see Supplementary Material). In general, the TET seems promising for future research as it did not only yield medium to very large effect sizes, but also shows on average—depending on the different conditions—comparable or higher values of empathic responsiveness to former studies using similar approaches in operationalizing empathy with standardized regression coefficients (Zaki et al., 2008, 2009; Morrison et al., 2016).

Overall, the findings of the current study support our first hypothesis, as women showed a significantly higher and facilitated (i.e., shorter response times) empathic response toward the friend compared to the enemy perspective. As proposed in the second hypothesis, even though the emotional closeness effect was apparent for both empathy components, the cognitive component was less affected (i.e., smaller difference in responsiveness as well as response facilitation) by the type of target person than the affective component. Independent of the target person, women showed overall significantly higher empathic responsiveness for the cognitive compared to the affective component. Regarding the modulatory effects of hormonal status, women in their early follicular phase exhibited significantly higher affective responsiveness toward the enemy than OC-users. Therefore, the third hypothesis was only partly supported as only affective empathic responsiveness toward the enemy and not affective empathy in general was modulated by hormonal status.

### Modulatory Effects of Emotional Closeness on Empathy Independent of Hormonal Status

Empathic abilities are crucial for successful social interactions (de Vignemont and Singer, 2006) and can be modulated factors such as familiarity (Engert et al., 2014; Meyer et al., 2015) and liking (Bucchioni et al., 2015). Whereas one study (Engert et al., 2014) observed increased empathic stress measured via elevated cortisol levels (i.e., affective component) for a loved person compared to a stranger, another study (Bucchioni et al., 2015) focused solely on the cognitive component of empathy for pain and also reported increased and facilitated perspective taking for a loved person compared to a stranger or a hated peer. In line with these studies, we found that regardless of empathy component, empathic responsiveness was significantly higher as well as facilitated (i.e., shorter response times) for the friend than for the enemy. Furthermore, even though cognitive compared to affective empathic responsiveness was significantly higher regardless of target person, the extent to which they differed was modulated by emotional closeness. Whereas the cognitive component was less affected by changes in emotional closeness, the affective component, as hypothesized, showed a significantly larger gap in empathic responsiveness toward the friend vs. enemy. Furthermore, affective compared to cognitive empathy was facilitated, as measured by smaller response times, for the friend perspective only. Overall, it seems that particularly the affective component of empathy is influenced by emotional closeness of the target person. Therefore, confirming the idea that at least to some extent empathy components work independently from each other (Zaki and Ochsner, 2012). This dissociation of the empathy components is also in line with the findings of a recent meta-analysis by Kogler et al. (2020) implicating the involvement of different neural networks in the generation of cognitive and affective empathy. Overall, it seems that the TET successfully replicates emotional closeness effects already found by previous studies. Furthermore, self-reported empathy correlated to some extent with the task-related empathy measures (see Supplementary Material). These observations implicate the TET as a valid, new measure of empathy. Finally, the TET did not only confirm previous findings, but also expanded upon them by showing in a direct comparison that affective empathy is more affected by the emotional closeness toward the target person than cognitive empathy.

### Modulatory Effects of Hormonal Status on Empathy

Regarding modulating effects of hormonal status, sex hormone related differences have been observed in various emotional processes (Montoya and Bos, 2017; Lundin et al., 2018; Lewis et al., 2019), including empathy components such as emotion recognition and affective responsiveness (Derntl et al., 2013). In line with former studies (Derntl et al., 2013; Radke and Derntl, 2016), hormonal status was associated to differences in the affective component of empathy. However, in this study only affective empathy toward the enemy varied with hormonal status. OC-users showed significantly less affective responsiveness toward the enemy than fNC women. Considering also the marginally higher affective responsiveness toward the friend in OC-users compared to fNC women, the lower affective empathy toward the enemy could be explained by a potentially hormone-influenced, elevated intergroup bias at the cost of the disliked person. However, this is pure speculation and needs to be scientifically proven with larger sample sizes in order to draw finite conclusions here.

Considering that OC use is associated with a 4-fold higher exposure to progestins compared to average cycle levels of endogenous progesterone in NC women (Lovett et al., 2017), it is likely that the high exposure to progestogens could have led to the relatively decreased empathic response toward the enemy. In NC women, progesterone levels were, however, previously positively associated with affective responsiveness (Derntl et al., 2013). Therefore, it might be possible that synthetic progestogens have different effects on affective responsiveness than endogenous progesterone. Nevertheless, this incongruency of findings could also be attributable to methodological differences of the studies using different types of target persons. Since the periovulatory women, who are characterized by high estradiol states, did not show any differences in empathic responses to the other two groups with low levels of estradiol, it seems that progesterone/progestogen rather than estradiol plays a modulatory role in female empathic processing. This is in line with a recent review by Sundstrom-Poromaa (2018) highlighting the relevance of progesterone in emotional processing of naturally cycling women. Even though we did not directly assess the association between testosterone and empathy, our findings are not in line with studies suggesting a negative association of testosterone with cognitive empathy (Nitschke and Bartz, 2020), as we found no differences between the OC and NC groups even though the OC group had significantly lower levels of testosterone.

A possible biological explanation for the higher susceptibility of the affective compared to the cognitive component of empathy could be a higher sex hormone sensitivity of brain regions consistently found to be involved in affective empathy [i.e., inferior frontal gyrus (IFG) and the posterior dorsomedial frontal gyrus (dmPFG)] as compared to the brain regions indicated for cognitive empathy [i.e., anterior dmPFG and supramarginal gyrus (Kogler et al., 2020)]. Indeed, according to Barth et al. (2015) sex hormone receptors are present in the frontal cortex including the dmPFG. The IFG has been repeatedly implicated to be modulated by endogenous as well as synthetic sex hormones (Silverman et al., 2011; Toffoletto et al., 2014), whereas the supramarginal gyrus has not yet been linked to hormone-induced alteration in activation nor its morphology. Silverman et al. (2011) linked especially the exposure to progestogens, which were used for a hormone-therapy, to a lower IFG activity. Since OC use exposes women to high levels of progestogens, it is likely that this exposure could also have neurobiological implications and possibly explain the behavioral differences found in the present study. Therefore, it seems worthwhile to also investigate potential neurobiological mechanisms leading to hormone-induced modulatory effects on empathic processes more closely to gain a better understanding of empathic processes in females.

Interestingly for both potential modulators (i.e., emotional closeness and hormonal status) cognitive empathy was significantly less modulated than affective empathy. Therefore, it seems that in this case cognitive empathy is less sensitive to modulators. As hypothesized, this could be due to its more rational nature by using reasoning (Einolf, 2012; Zaki and Ochsner, 2012), making it less sensitive if cognitive functioning is fully intact. Alternatively, the robustness could also be explained by generally high trait cognitive empathy skills of this female sample, as inferred by the self-reported scores for perspective taking. Nevertheless, considering the important role of affective empathy for prosocial behavior (Contreras-Huerta et al., 2020), the proneness of the affective empathy component to be influenced by factors such as emotional closeness and hormonal status, could have meaningful consequences for social functioning and interpersonal relationships. Therefore, these modulatory effects should be investigated more closely by including more levels of emotional closeness and a wider range of hormonal states in future studies.

### Limitations

The TET controls for other emotional processes such as the perception of non-verbal emotional signals confounding empathic processes due to the textual presentation of emotional situations and comprises the assessment of empathic responses in various situations including a range of positive as well as negative emotions, but there are also drawbacks to this task design. Due to time constraints of task length, positive and negative emotions were only built up from three emotions each, therefore not representing the whole spectrum of different emotions missing core emotions such as sadness. However, since we aimed for a balanced representation of positive and negative emotions to improve generalizability and a pre-study provided better validity for other emotions than sadness (see Supplementary Material for more information), this was a necessary step for this study’s design. Furthermore, the ability of participants to imagine the described scenarios was not measured. Differences in imagination skills but also in individual susceptibility to social desirability could have influenced the subjective ratings. Therefore, it would be advisable to account for these potential confounders either by questionnaires or by experimental tasks in the future.

Even though the sample size was comparable to former conceptually related studies (Derntl et al., 2013; Radke and Derntl, 2016; Dan et al., 2019; Gurvich et al., 2020), it could have been beneficial to increase statistical power by — next to having a larger sample size — using a within-subject design (Gonzales and Ferrer, 2016). However, the hormonal status groups were homogenous regarding various sociodemographic as well as neuropsychological parameters and were carefully selected. Therefore, results presented in this first investigation of the modulatory effect of hormonal status on empathic responsiveness toward different target persons with varying emotional closeness should be representative. Furthermore, to provide a more complete account of modulatory effects of hormonal status on female empathic responsiveness, future studies should also include hormonal states with high levels of both endogenous estradiol and progesterone, such as the luteal phase and pregnancy for instance. To disentangle progesterone and estradiol effects in naturally cycling women, studies with administration of progesterone or estradiol could be promising.

### Implications

The implications of this study are manifold. Firstly, empathic responsiveness toward a target person cannot be generalized to other target persons with varying levels of emotional closeness. Therefore, this study highlights the importance of including multiple target persons with varying degrees of emotional closeness in experimental paradigms to provide a more accurate and global assessments of empathic responsiveness. Secondly, female hormonal status seems to influence some empathic processes. Considering the steady and long-term use of OCs, the indication that OC use may negatively impact affective empathy toward target persons which are not very familiar is noteworthy considering its important role in social interactions (de Vignemont and Singer, 2006). Therefore, research on this topic is not only valuable for understanding potential non-conceptive side effects of OCs but can provide necessary information for women to make well informed contraceptive decisions. Furthermore, this study provides some evidence to support Christov-Moore et al. (2014) suggestion to account for hormonal status in studies examining sex differences to help resolving the present inconsistency in findings regarding experimental measures of empathy (Baez et al., 2017). Lastly, this study’s finding that the affective component of empathy might be more susceptible to different modulatory factors than the cognitive component could be of clinical relevance for treating empathic deficits present in some mental disorders by tailoring therapies to account for these modulatory influences (i.e., affective empathy training using a range of target persons with variable emotional closeness), as the affective component of empathy has been shown to be an important predictor of prosocial behavior not only in healthy people, but also across a range of different mental illnesses (Contreras-Huerta et al., 2020), and prosocial behavior plays an essential role in social functioning.

## Conclusion

Using the newly developed TET presenting textual descriptions of real-life situations, it became apparent that cognitive and affective empathy are differentially influenced by factors such as emotional closeness and hormonal status. In sum, cognitive empathy seems to be less sensitive than affective empathy to potentially modulating factors including emotional closeness and liking toward the target person, as well as hormonal status of a female perceiver. OC use was associated with less affective responsiveness toward a person with low emotional closeness compared to naturally cycling women in the early follicular phase. This first investigation of the influence of hormonal status on empathic responsiveness toward target persons with varying levels of emotional closeness highlights the importance for future research to shed more light on the role of hormonal status, and OC use in particular, in female empathic processing not only to understand the impact of sex hormones on empathy better, but also to, ultimately, allow women to make more informed contraceptive choices.

## Data Availability Statement

The datasets presented in this study can be found in online repositories. The names of the repository/repositories and accession number(s) can be found below: Mendeley: http://dx.doi.org/10.17632/2wxxyntrcv.2.

## Ethics Statement

The studies involving human participants were reviewed and approved by the Ethics committee of the Medical Faculty of the University Tübingen (331/2016BO2). The patients/participants provided their written informed consent to participate in this study.

## Author Contributions

A-CK and S-MW collected the data. A-CK performed the data analyses and wrote the manuscript. BD, DW, and IS helped with the methodological set-up. BD and DW were involved in the planning of data analysis and interpretation of data. A-CK and BD designed the study and supervised data collection. All authors contributed to the manuscript.

## Conflict of Interest

The authors declare that the research was conducted in the absence of any commercial or financial relationships that could be construed as a potential conflict of interest.
