# Supplementary material for: Friend vs. Foe: Cognitive and Affective Empathy in Women With Different Hormonal States
Source: Front Neurosci. 2021 Mar 8;15:608768. doi: 10.3389/fnins.2021.608768 (PMC7982725; doi:10.3389/fnins.2021.608768)
Supplement: Supplementary file 1 [file Table_1.DOCX]

**Methods**

**Sample description**

OC-users included 11 women using OCs containing second generation progestogens (i.e. androgenic progestogens), 10 women using OCs with fourth generation progestogens (i.e. anti-androgenic progestogens) and one woman using a third-generation progestogen (i.e. anti-androgenic progestogens). The minimum intake duration of the present OC was 6 months ranging to a maximum of about 7 years continued intake (i.e. 86 months), with a mean duration of 39.9 ± 20.8 months (i.e. 3.3 years ± 1.7 years). For the oNC group, NADAL hLH ovulation strips (nal von minden GmbH, Moers/Germany) were used to detect the luteinizing hormone (LH) surge predicting ovulation within 2 days (Palmer et al., 2007).

**Hormone analysis**

The used antibodies for these analyses included an anti-oestradiol monoclonal sheep antibody, a monoclonal mouse-anti-progesterone-antibody, a monoclonal sheep anti-testosterone-antibody and a monoclonal mouse Anti-SHBG antibody, respectively. The analytical sensitivity of the assays is 27.2pmol/l for oestradiol, 0.67 nmol/l for progesterone, 0.09 nmol/l for testosterone and 1.6 nmol/l for SHBG. For the intra-assay accuracy, the maximum coefficient of variation is 11.1% for oestradiol, 12.4% for progesterone, 8.5% for testosterone and 3.8% for SHBG. The reported overall variation of the assays is 13.3% for oestradiol, 12.7% for progesterone, 12.6% for testosterone and 6.5% for SHBG.

**Empathy Task**

**Pre-validation**. In a small pre-study (n = 12, only females), participants had to identify the emotion the described situation (n = 102) would most likely elicit. Only items with a rating concordance of 83.3% or higher regarding the identification of the emotional category within a forced choice paradigm (11 alternatives) were selected. In total, items for 10 different emotions were tested including pride, happiness, relief, sexual pleasure, gratefulness, disgust, anger, sadness, fear, and shame. For each positive and negative valence, the three emotions with the highest accuracy rates were chosen. In a second question, participants rated the intensity of the emotion felt in the described situation. Only items with ratings of the expected valence qualified for final selection. If more than six items per emotion had recognition accuracies higher than 80 percent, items with the highest intensity ratings were selected.

**Stimuli rating task**. In a rating task, the 62 recruited participants were asked to read and rate the textual descriptions of emotional scenes (in the self-perspective) according to their valence (from -50 = very negative to +50 = very positive), arousal (from -50 = very relaxed to +50 = very aroused) and dominance (from -50 = very out-of-control to +50 = very in control). Table S1 shows that there is a clear difference in valence between positive and negative emotions. The difference between positive and negative emotions was significantly smaller for arousal and dominance, with negative emotions being rated slightly more arousing and less dominant.

*Table S1: Mean raw scores (±standard deviation) of all ratings per valence of emotions (positive, negative) for valence, arousal and dominance ratings during the stimuli rating task*

|  | Valence | Arousal | Dominance |
| --- | --- | --- | --- |
| **Positive emotions**  **Negative emotions** | 32.58 (9.46) | 10.40 (14.06) | -16.73 (11.61) |
|  | -24.82 (7.25) | 15.31 (9.60) | -8.72 (12.07) |

**Correlational Analyses of self-reported and task-related empathy measures**

Pearson’s and Spearman’s correlations, depending on the data’s properties, were run to analyse relationships between self-reported empathy, measured by the interpersonal reactivity index and the task measures including empathic responsiveness (i.e. stand. regression coefficients) as well as response times. The trait empathy measured by the IRI was correlated with friend as well as enemy-related empathic responsiveness. The subscale perspective tasking was correlated with the cognitive empathy values and response times for the friend and enemy separately, whereas the subscale empathic concern was correlated to the affective empathy measures. Bonferroni-correction was applied separately for different IRI scales and regression coefficients versus response times.

**Results**

**Hormone profiles**

Bonferroni corrected post-hoc analyses of the main effect of estradiol (H(2)=49.86, *p*<.001, *_p_*η^2^=.46) indicated higher levels in the oNC group compared to both other groups (oNC vs OC: *p*<.001; oNC vs fNC: *p*<.01) and in the fNC compared to the OC group (*p*<.001). Progesterone (H(2) = 13.29, *p* = .001, *_p_*η^2^ = .18), on the other hand, did not differ between the NC groups (*p*=1.00), but was significantly lower in OC-users (fNC: *p*=.02; oNC: *p*=.002). Testosterone, (*F*(2,61)=15.95; *p*<.001, *_p_*η^2^=.21), was significantly lower in the OC group than in NC groups (OC vs fNC: *p*=.001; OC vs oNC: *p*<.001), while the NC groups had comparable testosterone levels (*p*=.28). Lastly, SHBG (H(2)=24.19, *p*<.001, *_p_*η^2^=.29) was significantly higher in OC-users compared to both NC groups (OC vs fNC: *p*=.001; OC vs oNC: *p*<.001), yet again there was no difference between the NC groups (*p*=1.00).

**Correlational Analyses of self-reported and task empathy measures**

*Empathic responsiveness*

Self-reported trait empathy was positively correlated to friend-related (*r_s_*=0.26, *p_1-tailed_*=.04) but not to enemy-related empathic responsiveness (*r_s_*=-0.05, *p*=.72). The perspective-taking scale did not correlate significantly with cognitive empathic responses towards the friend (*r_s_*=0.17, *p*=.38) nor the enemy (*r_s_*=0.22, *p*=.18). Empathic concern, on the other hand, showed a significant positive correlation with friend-related affective responsiveness (*r_s_*=0.31, *p*=0.03). The correlation for the enemy was non-significant (*r*=-.17, *p*=0.36).

*Response times*

There were no significant correlations between any IRI scales and respective response time measures (all |*r*| < .13, *p* ≥ .32).

**References**

Palmer, O.M., Grenache, D.G., and Gronowski, A.M. (2007). The NACB Laboratory Medicine Practice Guidelines for Point of Care Reproductive Testing. *Point of Care* 6(4)**,** 265-272. doi: 10.1097/poc.0b013e3180a02b57.
